# The role of ubiquitination in the pathogenesis of atrial fibrillation: mechanisms and therapeutic implications

**DOI:** 10.3389/fphar.2026.1784147

**Published:** 2026-03-26

**Authors:** Zhang Runtian, Guo Xing, Shi You, Shen Zimeng, Han Wenqiang, Cui Likun, Miao Qingrun, Zhong Jingquan

**Affiliations:** 1 Department of Cardiology, State Key Laboratory for Innovation and Transformation of Luobing Theory, Key Laboratory of Cardiovascular Remodeling and Function Research, Chinese Ministry of Education, Chinese National Health Commission and Chinese Academy of Medical Sciences, Qilu Hospital of Shandong University, Jinan, China; 2 Department of Cardiology, Qilu Hospital of Shandong University (Qingdao), Qingdao, China; 3 School of Basic Medical Sciences, Cheeloo College of Medicine, Shandong University, Jinan, Shandong, China

**Keywords:** atrial fibrillation, autophagy, calcium dysregulation, ferroptosis, fibrosis, inflammation, oxidative stress, small molecule drugs

## Abstract

Atrial fibrillation (AF) is the most prevalent cardiac arrhythmia, defined by rapid and irregular atrial activation. Despite advances in AF treatment, AF remains a leading cause of cardiovascular morbidity and mortality worldwide. Emerging evidence demonstrates that ubiquitination, a dynamic post-translational modification, acts as a central regulator of AF pathogenesis by modulating inflammatory signaling, calcium dysregulation, ferroptosis, oxidative stress, and autophagy. This review systematically explores the multifaceted roles of ubiquitin-related enzymes (E3 ligases and deubiquitinating enzymes, DUBs) in AF pathogenesis, with an emphasis on their interactions with core molecular pathways. Furthermore, we discuss potential therapeutic strategies targeting the ubiquitin-proteasome system, and provide a framework for future translational research in AF.

## 1 Introduction

Atrial fibrillation (AF) is a condition characterized by rapid activity in different regions of the atria, which overrides the normal sinoatrial node’s control of heart rhythm. This results in rapid and irregular atrial activity and, instead of contracting, the atria only quiver (Nattel, 2002). It is the most common arrhythmia and significantly impacts cardiovascular disease morbidity and mortality (Cheng et al., 2024). In 2023, the absolute prevalence of AF in the Asia-Pacific region, which holds 60% of the global population, was estimated to be approximately 80 million (Wong et al., 2024). This underscores the necessity for early detection and the urgent development of more effective AF management strategies, highlighting the urgent need for a deeper understanding of AF pathogenesis.

The pathogenesis of AF is diverse. Atrial remodeling is an important pathological manifestation of atrial fibrillation, which refers to the structural, electrophysiological, and molecular-level adaptive or pathological changes in the atria under chronic pathological stimuli or persistent electrical activity abnormalities caused by AF itself. It can be subdivided into electrical remodeling, structural remodeling. There is a vicious cycle relationship between atrial remodeling and AF. On one hand, atrial remodeling sustains and exacerbates AF (Blaauw and Crijns, 2007). On the other hand, AF induces atrial remodeling (Franz et al., 1997).

Oxidative stress, fibrosis, inflammation, and other factors can participate in the occurrence and development of atrial fibrillation by regulating atrial remodeling (Sagris et al., 2021; Brundel et al., 2022). These findings suggest that the occurrence of AF results from the combined effects of multiple factors, involving the interaction of various biological processes. Recent studies point to aberrant molecular regulation, particularly protein post-translational modifications (PTMs), as one of the core mechanisms in AF progression (Oh et al., 2020). For example, protein phosphorylation and lysine 2-hydroxyisobutyrylation have been demonstrated to be involved in the initiation and progression of atrial fibrillation. (Safabakhsh et al., 2022; Hou et al., 2025). Ubiquitination, as one of the most complex PTM systems in eukaryotes, still has significant knowledge gaps regarding its specific regulatory network in AF pathogenesis.

Ubiquitin (Ub) is a highly conserved 76-amino acid polypeptide that is covalently attached to one or more lysine residues of cellular proteins through a conserved enzymatic cascade reaction known as ubiquitination. Ubiquitin itself contains seven lysine residues, each of which can be further conjugated to the carboxyl-terminus of another ubiquitin molecule, forming polyubiquitin chains.

Ubiquitination is a crucial post-translational modification involved in regulating nearly all eukaryotic cellular processes (Yau and Rape, 2016). This regulation is precisely balanced by the action of the ubiquitin-conjugating enzyme system and deubiquitinating enzymes (Oh et al., 2018). The ubiquitin-conjugating enzyme system consists of three enzymes: Ub-activating enzyme (E1), Ub-conjugating enzyme (E2), and Ub ligase (E3), which link Ub to substrate proteins via isopeptide bonds to regulate substrate degradation (Schulman and Harper, 2009; Ye and Rape, 2009; Zheng and Shabek, 2017). Like many biological processes, ubiquitination is reversible; deubiquitinating enzymes (DUBs) can reverse this process by hydrolyzing the isopeptide bond, releasing Ub from ubiquitinated substrates, making it ideally suited for regulatory control. Ubiquitin or polyubiquitin chains act as three-dimensional signals that can be recognized by unique Ub-binding proteins (UBPs) in a conformation-specific manner to ensure robust and faithful signal transduction in eukaryotic cells (Husnjak and Dikic, 2012). Thus, the ubiquitination process can be analogized to a dynamic message: written by the ubiquitin-conjugating enzyme system, read by UBPs, and erased by DUBs (Zheng et al., 2023).

The human proteome includes 2 ubiquitin E1 enzymes, approximately 50 E2s, 600 E3s, 90 DUBs, and 20 types of Ub-binding domains (UBDs) (Bhoj and Chen, 2009). Furthermore, there are hundreds of UBPs comprising at least 20 UBD families that mediate selective recognition in Ub signaling (Husnjak and Dikic, 2012). Ubiquitinase and deubiquitinase exert extensive and pervasive effects on eukaryotic cell functions by regulating protein stability and degradation, which reinforces its relevance to cardiovascular physiology and pathology.

Specifically, ubiquitination modulates not only substrate turnover but also protein subcellular localization, enzymatic activity, protein-protein interactions, and the efficiency of intracellular signal transduction cascades (Yang X. et al., 2025). For example, ubiquitin-specific protease 38 (USP38) is a deubiquitinase capable of regulating inflammation, cell proliferation, migration, and iron deposition through the stabilization of target proteins such as P53 and HMX3 (Gong et al., 2023a; Wang et al., 2023; Gong et al., 2025). Tripartite motif-containing protein 21 (TRIM21), an E3 ubiquitin ligase, regulates oxidative stress and immune responses by promoting the degradation of P62 and Sohlh2 (Yang P. et al., 2025; Zhang et al., 2023). These diverse regulatory effects enable the ubiquitin system to govern core cellular processes, including cell proliferation, differentiation, inflammatory responses, oxidative stress adaptation, and programmed cell death—all of which are critical to cardiac homeostasis and disease progression (Zeng et al., 2025; Powell et al., 2012).

**FIGURE 1:**
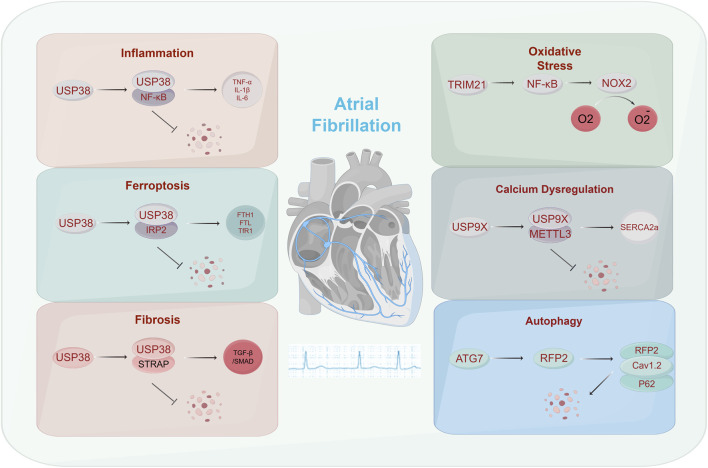
The mechanism of action of ubiquitination on atrial fibrillation. Ubiquitination can affect atrial fibrillation through various physiological phenomena, such as inflammation, ferroptosis, fibrosis, oxidative stress, calcium dysregulation, and autophagy.

Growing evidence indicates that ubiquitination plays a key role in cardiovascular diseases such as cardiac hypertrophy, myocardial ischemia-reperfusion injury, arrhythmias, diabetic cardiomyopathy, and atherosclerosis (Yan et al., 2019; Hu et al., 2018; Liu et al., 2021; Shu et al., 2022). Recent studies demonstrate its critical role in the initiation and progression of atrial fibrillation. Ubiquitination participates in atrial remodeling through the dynamic regulation of ion channel stability, inflammasome activation, and programmed cell death pathways. However, these findings are often fragmented. In this review, we aim to systematically integrate recent breakthroughs by focusing on the roles of ubiquitination in six key aspects of AF pathogenesis: inflammation, calcium dysregulation, ferroptosis, oxidative stress, autophagy and fibrosis (Figure 1; Table 1). By constructing a multidimensional map of the E3-DUB-substrate regulatory axis, we intend to provide a theoretical framework for precise therapeutic strategies.

**TABLE 1:** Ubiquitinating and deubiquitinating enzymes regulating key substrates in atrial fibrillation.

Substrate	Physiological function	Effect on AF	Ubiquitinating enzyme/Deubiquitinating enzyme	Regulatory mechanism
NF-κB	Increasing inflammatory cytokine release (TNF-α, IL-1β)	Promoting inflammation	USP38 (DUB)	USP38 promotes NF-κB homeostasis
METTL3	Regulating calcium homeostasis	Regulating calcium dysregulation	USP9X (DUB)	USP9X promotes METTL3 homeostasis
RYR2	Regulating Ca^2+^ release from the sarcoplasmic reticulum	Regulating calcium dysregulation	UBE2C (E2)/TRIM24 (E3)	UBE2C/TRIM24 promotes ubiquitination of RYR2
Nox2	Promoting ROS production	Regulating Oxidative Stress	TRIM21 (E3)	TRIM21 regulates Nox2 via the NF-κB pathway
Cav1.2	Mediating myocardial calcium ion influx	Regulating atrial effective refractory period	RFP2(E3)	RFP2 mediates selective autophagy-dependent protein degradation
IRP2	Inducing iron overload and triggers ferroptosis	Regulating ferroptosis	USP38 (DUB)	USP38 promotes IRP2 homeostasis
STRAP	Regulating TGF-β/SMAD signaling pathway	Regulating Fibrosis	USP38 (DUB)	USP38 promotes STRAP homeostasis

## 2 Inflammation

Inflammation involves the release of cytokines, the production of reactive oxygen species (ROS), and the activation of enzyme systems for tissue degradation and repair (Friedrichs et al., 2011). Inflammatory pathways participate in atrial structural remodeling through multiple mechanisms. They not only regulate ion channel function and cardiomyocyte ion homeostasis but also disrupt the homogeneity of the atrial tissue extracellular matrix and jointly drive atrial tissue remodeling by promoting pathological fibrosis processes. This makes inflammation contribute to atrial remodeling, including adaptive or maladaptive changes in the structure, function, and electrophysiological properties of the atria (Hu et al., 2015). Collectively, this evidence underscores the important pathophysiological role of inflammation in the occurrence of AF.

It is noteworthy that NF-κB, a key family of transcription factors regulating inflammatory responses, promotes the transcription of many inflammatory cytokines during atrial remodeling (Sh et al., 2019). NF-κB refers to dimeric transcription factors, including members of the Rel family of DNA-binding proteins. Five members of the mammalian Rel family are known: RelA (p65), RelB, c-Rel, NF-κB1 (p50), and NF-κB2 (p52) (Baldwin, 1996; Ghosh et al., 1998). In the absence of external stimuli, NF-κB exists in the cytoplasm as homo- or heterodimers bound to IκB proteins, which inhibits its nuclear localization and DNA-binding function (Whiteside and Israël, 1997). The NF-κB pathway is closely linked to AF. It is activated in atrial fibrillation and can influence AF progression by regulating inflammation, atrial remodeling, and electrical remodeling (Gao and Dudley, 2009; Li et al., 2020; Wang H. et al., 2020; Yu et al., 2024; Kong et al., 2022).

In the cardiovascular system, USP38 is involved in pathological cardiac remodeling and cardiac arrhythmias through ubiquitination-dependent mechanisms (Gong et al., 2023a; Gong et al., 2025; Xiao et al., 2024a; Chen et al., 2026). Zheng et al., using a mouse model of aortic banding (AB) surgery and an angiotensin II (Ang II)-stimulated HL-1 cell model, first revealed the critical role of USP38 in pressure overload-induced AF (Xiao et al., 2023). USP38 showed a time-dependent upregulation in the AB model mice and Ang II-treated HL-1 cells. The researchers generated cardiac-specific knockout (cardiomyocyte conditional knockout) USP38 (USP38^cko^) mice. Compared to wild-type mice, the AB-induced shortening of the effective refractory period and the pressure overload-induced AF vulnerability and duration were significantly improved in USP38^cko^ mice, demonstrating that USP38 increases atrial fibrillation vulnerability. Mechanistically, cardiomyocyte-specific knockout of USP38 significantly ameliorated pressure overload-induced atrial structural remodeling (reducing left atrial enlargement, lowering collagen deposition) and electrical remodeling, whereas USP38 overexpression exacerbated these pathological changes.

Compared to wild-type mice, USP38^cko^ mice exhibited lower levels of inflammatory cytokines (such as TNF-α, IL-1β, IL-6) and p-NF-κB (Ser536) in serum and atrial tissue. While USP38 overexpression further upregulated p-NF-κB levels. Concurrently, *in vitro* experiments also demonstrated that USP38 could upregulate p-NF-κB levels. This indicates that USP38 may regulate the level of inflammation in AF by affecting p-NF-κB levels.

To investigate the specific pathway through which USP38 affects inflammation and AF susceptibility, researchers confirmed the USP38-NF-κB interaction mechanism through multidimensional experiments: Immunofluorescence showed significant co-localization of USP38 and NF-κB in Ang II-stimulated HL-1 cells. Co-immunoprecipitation (Co-IP) confirmed that USP38 antibody could specifically precipitate the NF-κB complex, and *vice versa*. More importantly, ubiquitination assays showed that USP38 knockdown significantly increased NF-κB ubiquitination levels, while its overexpression decreased NF-κB ubiquitination. This demonstrates that USP38, by directly binding to NF-κB and reducing its ubiquitination level, promotes phosphorylation at Ser536 and nuclear translocation, thereby driving the expression of inflammatory factors such as TNF-α, IL-1β, and IL-6. Simultaneously, the upregulated NOD-like receptor protein 3 (NLRP3) inflammasome can enhance NF-κB pathway activity, forming a NF-κB/NLRP3 positive feedback loop. Moreover, injection of NF-κB inhibitor PDTC can completely block atrial electrophysiological abnormalities caused by USP38 overexpression, further confirming that USP38 regulates atrial remodeling through the NF - κ B/NLRP3 axis.

These research results demonstrate the important role of the USP38/NF-κB/NLRP3 axis in regulating the progression of adverse atrial remodeling induced by pressure overload. Consistently, the role of the NF-κB/NLRP3 pathway in promoting AF progression has been well-established in other studies (Kong et al., 2022; Xiao et al., 2023; Ren et al., 2022). In another study, Yang et al. reported that cardiac-specific knockout of USP38 attenuated left atrial inflammation and reduced AF susceptibility following myocardial infarction. Mechanistically, cardiac-specific ablation of USP38 suppressed transforming growth factor-β-activated kinase 1 (TAK1) phosphorylation and subsequent activation of the NF-κB signaling pathway, whereas cardiac-specific overexpression of USP38 exerted the opposite effects (Gong et al., 2023b). These findings indicate that, beyond directly modulating the ubiquitination status of NF-κB, USP38 may also regulate AF susceptibility by promoting TAK1 phosphorylation and thereby activating the NF-κB signaling cascade. Therefore, USP38 may serve as a novel therapeutic target for pressure overload-induced AF.

## 3 Calcium dysregulation

The pathological remodeling in atrial fibrillation (AF) is closely related to calcium homeostasis imbalance. In recent years, substantial evidence has indicated that abnormal Ca^2+^ handling plays a key role in the initiation and maintenance of AF (Hove-Madsen et al., 2004; Dobrev, 2010; Neef et al., 2010). Abnormal release and reuptake of calcium transients in atrial cardiomyocytes can lead to disordered electrical activity and structural remodeling, forming a vicious cycle of AF begets AF (Liao et al., 2021). Recent studies have found that ubiquitination modifications, by dynamically regulating the stability, function, and localization of calcium-handling proteins, serve as a key molecular bridge connecting calcium dysregulation and the progression of cardiovascular diseases (Cheng et al., 2025). This section will systematically elaborate on the specific mechanisms by which ubiquitination influences AF through calcium homeostasis imbalance.

A recent study exploring the role of METTL3 in atrial cardiomyocytes in AF uncovered another mechanism by which ubiquitination contributes to AF progression. Researchers found that METTL3, a methyltransferase, was downregulated in left atrial appendage samples from AF patients (Shi et al., 2025). Concurrently, after knocking down METTL3 in primary neonatal mouse atrial myocytes (NMAMs), calcium transients induced by 1 Hz electrical stimulation showed increased amplitude and shortened decay time. Experiments in adult mouse atrial myocytes (AMAMs) also showed significantly accelerated calcium transient kinetics, suggesting that METTL3 deficiency leads to abnormal calcium handling function in atrial myocytes. In-depth mechanistic analysis revealed that METTL3 downregulates the expression of protocadherin PCDHGA10 through epigenetic regulation, thereby inhibiting the activity of the sarcoplasmic reticulum calcium pump SERCA2a, reducing sarcoplasmic reticulum calcium load, and ultimately decreasing AF susceptibility.

To investigate the mechanism of METTL3 downregulation in AF models, researchers examined the expression levels of CREB1 protein (a transcription factor associated with AF occurrence) and its phosphorylated form (p-CREB1). The results showed that both were significantly reduced in left atrial appendage samples from AF patients. Meanwhile, knocking down CREB1 in HL-1 cells resulted in a significant decrease in METTL3 protein levels, while its mRNA expression was unaffected, suggesting a post-transcriptional regulatory mechanism.

Further mechanistic studies showed that after knocking down CREB1 in HL-1 cells, the mRNA and protein expression of Ubiquitin-specific peptidase 9 X-linked (USP9X) were significantly downregulated, a phenomenon consistent with the characteristics of AF patient samples. USP9X is a deubiquitinating enzyme implicated in the regulation of cell apoptosis, migration, and DNA damage repair (Meng et al., 2023). In the cardiovascular system, USP9X modulates the ubiquitination of specific substrates, including SR-A1 and TFEB, thereby influencing the development and progression of atherosclerosis and abdominal aortic aneurysm. Dual-luciferase reporter assays confirmed that CREB1 directly regulates the transcriptional activity of USP9X (Wang B. et al., 2022; Chen J. et al., 2025).

More importantly, knocking down USP9X in HL-1 cells led to a significant decrease in METTL3 protein levels, accompanied by increased METTL3 ubiquitination, indicating that USP9X maintains METTL3 protein stability through deubiquitination. The study found that the CREB1/USP9X/METTL3/PCDHGA10 axis in atrial cardiomyocytes plays a key role in AF development by affecting calcium handling. Consistent with these findings regarding calcium handling, Meng et al. further demonstrated that USP38 promotes calcium homeostasis disruption, which in turn exacerbates atrial electrical instability under pathological stress (Meng et al., 2025).

Furthermore, targeting the ubiquitination of Ryanodine Receptor 2 (RYR2) holds great potential for alleviating calcium dysregulation in AF. As the major calcium release channel on the sarcoplasmic reticulum, RYR2 plays a critical role in the development of calcium overload by regulating Ca^2+^ release from the sarcoplasmic reticulum into the cytoplasm. RYR2-mediated Ca^2+^ leakage constitutes a key mechanism underlying the pathogenesis of atrial fibrillation, and pharmacological interventions targeting RYR2 may represent effective therapeutic strategies for this arrhythmia (Gillis and Dobrev, 2022; Dridi et al., 2020; Kim et al., 2023). Although direct evidence linking RYR2 ubiquitination to atrial fibrillation remains lacking, Wang et al. demonstrated that ubiquitination and degradation of Integrin β1D contribute to altered RYR2 function, thereby promoting ventricular tachycardia (Wang Y. et al., 2020). Furthermore, Duan et al. reported that UBE2C, an E2 ubiquitin-conjugating enzyme, facilitates the ubiquitination and degradation of RYR2 (Zhai et al., 2022). In addition, TRIM24 has been shown to modulate RYR2 expression and activity at the tissue level, thereby disrupting intracellular calcium homeostasis (Neu et al., 2025). This reveals an additional mechanism through which ubiquitination promotes AF: by dysregulating calcium handling.

## 4 Ferroptosis

Recent studies have revealed that dysregulation of cell death pathways is a key mechanism driving atrial fibrillation. In addition to classical apoptosis and necrosis, ferroptosis—an iron-dependent, lipid peroxidation-driven novel form of programmed cell death—is increasingly gaining attention in cardiovascular diseases (Liu L. et al., 2023; Fang X. et al., 2023). Recent studies suggest that the occurrence of atrial fibrillation may be associated with ferroptosis activation. Experimental data show that the ferroptosis inhibitor ferrostatin-1 significantly reduces AF susceptibility in various pathological models (such as sepsis, obesity, and excessive ethanol intake) (Kong et al., 2022; Fang et al., 2021; Dai et al., 2022), suggesting that targeting the ferroptosis pathway may provide a new direction for AF intervention.

Zheng et al. demonstrated that USP38 plays a key regulatory role in the development of diabetes-related atrial fibrillation (Xiao et al., 2024b). In high glucose-stimulated *in vivo* and *in vitro* models, USP38 expression was significantly upregulated. Overexpression of USP38 in diabetic mice significantly shortened the PR interval, accompanied by increased AF susceptibility and duration.

Simultaneously, USP38 overexpression disrupted cardiomyocyte calcium homeostasis, accelerated the structural remodeling of the gap junction protein connexin 43 (Cx43), and, by inducing iron overload and oxidative stress, aggravated ferroptosis in the atrial tissue of diabetic mice, ultimately increasing the susceptibility of diabetic mice to atrial fibrillation. Conversely, USP38 knockout significantly reduced AF susceptibility and effectively inhibited the ferroptosis process.

Building on this, the study revealed the specific mechanism of USP38-regulated ferroptosis through multidimensional experiments. Immunofluorescence co-localization and bidirectional Co-IP confirmed a direct interaction between USP38 and iron Regulatory Protein 2 (IRP2) in HL-1 cells. Truncation experiments showed that the ΔD1 domain (Δ1-316 aa) of IRP2 is the key region mediating this interaction. Ubiquitination analysis indicated that USP38 overexpression significantly decreased IRP2 ubiquitination levels, while an enzyme-dead mutant had no such effect, confirming that USP38 stabilizes IRP2 protein through deubiquitination.

To explore the role of iron overload in diabetes-induced AF, the study employed the iron chelator deferoxamine (DFO) for intervention experiments. The results showed that DFO treatment significantly reversed the pathological phenotypes caused by USP38 overexpression, markedly reducing the AF induction rate in the diabetic model mice, while also lowering myocardial iron content and oxidative stress levels, confirming that iron overload is a key downstream effector mechanism of the USP38-IRP2 axis.

The study elucidates the molecular pathway: High glucose/USP38/IRP2 stabilization/Ferroptosis/AF susceptibility. This not only provides a new mechanistic explanation for diabetes-related AF but also suggests that targeting the USP38/IRP2 axis or the ferroptosis process may become an innovative strategy for preventing and treating metabolic atrial fibrillation. Notably, ubiquitination modifications, by precisely regulating the stability of key molecules in ferroptosis, act as a molecular switch in the AF process, a finding that introduces a new paradigm for research on epigenetic regulation in cardiovascular diseases.

## 5 Oxidative stress

Atrial remodeling is an important pathological basis during the initiation, maintenance, and recurrence of atrial fibrillation. Studies have established that atrial remodeling is a major factor in the onset of AF following myocardial infarction (MI) (Miyauchi et al., 2003).

Liu et al.'s study first revealed that the E3 ubiquitin ligase TRIM21 participates in the occurrence and development of post-myocardial infarction atrial fibrillation by regulating the oxidative stress pathway. In an MI mouse model, TRIM21 knockout significantly improved atrial remodeling: reduced atrial fibrosis area, smaller left atrial diameter, and decreased cardiomyocyte apoptosis rate. Oxidative stress and inflammation are closely related to atrial remodeling. TRIM21 deficiency suppressed oxidative stress and inflammation in the left atrium (LA) post-MI. In HL-1 cells, TRIM21 overexpression exacerbated oxidative stress and apoptosis. Molecular mechanism studies showed that TRIM21 overexpression, by activating the NF-κB signaling pathway, upregulates NADPH oxidase expression, promotes increased ROS generation, and ultimately leading to increased apoptosis rate in HL-1 cells (Liu et al., 2023a).

To investigate the role of ROS, the inhibitor NAC was employed. NAC attenuated DNA damage and the pro-apoptotic effects induced by TRIM21 overexpression and reversed the TRIM21 overexpression-induced decrease in Cx43 expression.

NADPH oxidase is a major source of ROS in the cardiovascular system, and the main subtype expressed in human atria is Nox2 (Kim et al., 2005). The study found that TRIM21 can induce apoptosis and oxidative stress and reduce Cx43 expression in atrial cardiomyocytes, at least partly through the upregulation of Nox2.

Important evidence indicates that Nox2 can be regulated by NF-κB (Wang et al., 2019). The study further discovered the mechanism that TRIM21 promotes oxidative stress and Nox2 expression by activating the NF-κB pathway (Liu et al., 2023b). Given that TRIM21 mediates oxidative stress-associated atrial remodeling after myocardial infarction, which constitutes a critical driver for the initiation and progression of AF, TRIM21 may act as a novel regulator of AF via ubiquitination-dependent control of oxidative stress.

## 6 Autophagy

Autophagy is a highly regulated bulk degradation pathway that sequesters a portion of damaged proteins and organelles to maintain cellular homeostasis, especially in long-lived cells (Mizushima and Komatsu, 2011). Ubiquitination plays an important role in the electrical remodeling, structural remodeling, and metabolic imbalance of atrial fibrillation by precisely regulating the initiation, substrate recognition, and degradation processes of autophagy.

Yuan et al. (2018) found that autophagy activity was significantly enhanced in the left atrial appendage tissues of patients with persistent AF and verified this in animal models.

The study validated the molecular mechanism of the ubiquitination-autophagy axis regulating Cav1.2 channel degradation through multidimensional experiments. Knockdown of ATG7 effectively inhibited atrial autophagy activation during AF, significantly restored the shortening of the atrial effective refractory period (AERP), and reduced the incidence and duration of RAP-induced AF. Previous studies have demonstrated that downregulation of calcium channels, particularly Cav1.2, is a well-established feature of AF pathogenesis. The study showed that the autophagy model induced by ATG7 overexpression led to decreased Cav1.2 expression. Further Co-IP found that LC3B co-immunoprecipitated with Cav1.2 under normal conditions, indicating interaction between autophagosomes and the ICa,L α subunit. This demonstrated that activated autophagy induces the recruitment of Cav1.2 to LC3B-positive autophagosomes, thereby promoting the selective degradation of Cav1.2.

At the ubiquitination level, treatment of rapidly paced HL-1 cells with PYR-41—a selective inhibitor of the E1 ubiquitin-activating enzyme—markedly blocked Cav1.2 protein degradation, confirming that ubiquitination acts as an essential signal for the autophagy-dependent degradation of Cav1.2. In the pacing model, the expression of Ret Finger Protein 2 (RFP2), an E3 ubiquitin ligase, was significantly upregulated, and this increase was reversed upon ATG7 knockdown. Mechanistically, RFP2 mediates the ubiquitination of Cav1.2; the canonical autophagy receptor p62 then recognizes ubiquitinated Cav1.2 and targets it to LC3B-positive autophagosomes, ultimately leading to the selective degradation of Cav1.2. Co-IP assays verified that the binding affinity between p62 and Cav1.2 was markedly enhanced under AF conditions, accompanied by an increased colocalization rate between LC3B-positive autophagosomes and Cav1.2—whereas ATG7 knockdown exerted the opposite effects. Furthermore, chloroquine treatment effectively reversed Cav1.2 degradation, further validating the critical role of this ubiquitin–autophagy regulatory pathway in this process.

Lin et al. found that FOXO3a induces myocardial fibrosis by upregulating mitophagy (Bravo-San Pedro et al., 2017). Mitophagy is a selective intracellular degradation process that helps eliminate dysfunctional mitochondria and maintain mitochondrial homeostasis. The study demonstrated that FOXO3a plays a role in mediating myocardial fibrosis (MF) and ultimately leads to myocardial fibrosis through the PINK1/Parkin pathway (Lin et al., 2024). The PINK1/Parkin signaling pathway has been widely reported to participate in mitophagy. When mitochondrial membrane potential is lost, PINK1 accumulates on the outer mitochondrial membrane and stimulates the recruitment and activation of the E3 ubiquitin ligase Parkin (Ashrafi and Schwarz, 2013). Activated Parkin is responsible for conjugating ubiquitin molecules to adaptor proteins (e.g., sequestosome-1), thereby amplifying the mitophagy signal (Liu et al., 2023b).

The studies discussed above demonstrate that ubiquitination plays a critical role in regulating autophagy. At the same time, it should also be noted that autophagy plays a dual-edged role in AF; moderate autophagy may clear damaged mitochondria or abnormal protein aggregates, but its protective function in AF is masked by overactivated degradation pathways.

## 7 Fibrosis

AF is a prevalent arrhythmia closely associated with atrial fibrosis, notably, fibrosis is increasingly recognized as a key prognostic factor for the development of AF and its related complications, including stroke (Goette et al., 2024; Nattel, 2023; Miyauchi et al., 2023; Li M. et al., 2022; Li Z. et al., 2022). Fibrosis creates a substrate for AF through structural, electrical, and functional remodeling. It can be both a cause and a consequence of AF, and once established, it accelerates AF progression (Schotten et al., 2025). Atrial fibrosis is driven by multiple pathophysiological processes, with the TGF-β/SMAD signaling pathway serving as a key mediator. Activated in atrial fibrillation, the TGF-β/SMAD pathway contributes to the initiation and progression of atrial fibrillation primarily by promoting atrial fibrosis (Luo et al., 2024; Chen C. et al., 2025). Recently, Meng et al. revealed a crucial role for ubiquitination in regulating AF susceptibility through fibrosis (Meng et al., 2025).

AF occurs at a markedly higher incidence in patients with chronic kidney disease (CKD) than in healthy individuals, a phenomenon that may be attributed to CKD-driven AF promotion via the inflammasome pathway (Guo et al., 2019; Song et al., 2023). Researchers established a murine CKD model and observed that the AF inducibility rate was significantly elevated compared with the sham-operated group, accompanied by upregulated USP38 expression in atrial tissues (Meng et al., 2025). To explore the functional role of USP38, USP38^CKO^ mouse model was generated. Results demonstrated that USP38 ablation significantly attenuated CKD-induced atrial fibrosis, left atrial dilation, and AF susceptibility, whereas USP38 overexpression exacerbated these pathological alterations. Notably, although CKD mice exhibited significantly shortened AERP, neither USP38 knockout nor overexpression significantly modified AERP shortening. These findings indicated that USP38 modulates CKD-associated AF vulnerability primarily by regulating atrial fibrosis rather than by directly altering AERP. Mechanistically, molecular docking, Co-IP, and ubiquitination assays confirmed that USP38 interacts with STRAP and preserves its protein stability through deubiquitination. This event further activates the canonical TGF-β/SMAD fibrotic signaling pathway, thereby enhancing the expression of fibrotic markers including collagen I/III and α-SMA. Furthermore, STRAP knockdown was shown to reverse the profibrotic and proarrhythmic effects induced by USP38 overexpression.

Other ubiquitin ligases and deubiquitinases involved in cardiac remodeling also warrant further investigation. WW domain containing E3 ubiquitin protein ligase 1(WWP1) is a ubiquitin ligase that has been previously reported to degrade TβR1 and Smad2, thereby negatively regulating the TGF-β signaling pathway (Zhi and Chen, 2012). Tao et al. demonstrated that WWP1 is downregulated in atrial fibrillation and can attenuate atrial fibrosis by modulating the TGF-β signaling pathway (Tao et al., 2018). Similarly, TNF receptor-associated factor 6 (TRAF6), an E3 ubiquitin ligase, has been reported to be upregulated in atrial fibrillation (Liu et al., 2023b). TRAF6 has been demonstrated to promote atrial fibrosis in this condition and is also implicated in atrial fibrosis in patients with rheumatic heart disease who develop postoperative atrial fibrillation (Gu et al., 2012; Zhang et al., 2017). However, whether TRAF6 modulates atrial fibrosis via ubiquitination remains to be fully elucidated. In addition, WW domain containing E3 ubiquitin protein ligase 2(WWP2) modulates cardiac remodeling and myocardial fibrosis by mediating the ubiquitination of IRF7 in monocytes and macrophages, as well as by regulating PARP1 in cardiomyocytes (Zhang et al., 2020; Chen et al., 2022).

## 8 Future perspectives

RNA sequencing (RNA-Seq) is a crucial tool for investigating transcriptomic changes in disease models. In an RNA-Seq study, Thomas and colleagues recruited 5 patients with sinus rhythm and 5 patients with permanent atrial fibrillation, and performed RNA sequencing on their left and right atria to explore gene expression differences under AF conditions (Thomas et al., 2019). The results showed a total of 239 differentially expressed genes in the left and right atrial appendage. The significantly downregulated expression of the KBTBD13 gene in the right atrial appendage caught our attention. KBTBD13 is a substrate adapter for Cullin-3, a ubiquitin ligase that mediates ubiquitination (Sambuughin et al., 2012). KBTBD13 is highly expressed in cardiac muscle (Sambuughin et al., 2010). Mutations in KBTBD13 are associated with Nemaline Myopathy type 6 (NEM6), a type of nemaline myopathy (Sambuughin et al., 2012). Researchers constructed pedigrees of 3 families with KBTBD13 mutations, and 8% of the total 65 patients were complicated with atrial fibrillation (de Winter et al., 2022). This suggests that KBTBD13 may influence AF through its ubiquitination-regulating function, providing a potential target for subsequent AF treatment. Although no studies have linked other differentially expressed genes to AF to date, they may be involved in the pathogenesis and progression of AF by affecting key pathological processes such as inflammation and fibrosis, which requires further investigation (Goyenechea et al., 2009; Gao, 2025).

In cancer therapy, numerous small-molecule inhibitors targeting various components of the ubiquitin-proteasome system (UPS)—including the proteasome, E1/E2 enzymes, E3 ligases, and DUBs—have been developed, and their anti-tumor efficacy is under extensive evaluation (Deng et al., 2020). However, the therapeutic targeting of the ubiquitination network for atrial fibrillation using small-molecule inhibitors is still in its infancy and has not yet advanced to clinical development or application. As described above, the UPS plays a crucial role in the pathophysiology of AF. The ubiquitination regulatory network in AF provides abundant targets for the development of novel therapeutic strategies for AF and offers a solid theoretical foundation for the translational application of small-molecule drugs. Developing more specific targeted molecules, particularly those targeting UPS components specifically activated under AF conditions, is an extremely promising direction (Table 2).

**TABLE 2:** Small molecule drugs and their targets related to ubiquitination in atrial fibrillation.

Small molecule inhibitor	Target site	Effect on AF	Proposed mechanisms	References
LDN-57444	UCHL1	Alleviate atrial fibrillation	Regulating inflammation and fibrosis	Bi et al. (2020), Lei et al. (2020), Gong et al. (2021), Zhang J. et al. (2021)
P22077	USP7	Alleviate atrial fibrillation	Regulating inflammation, fibrosis and oxidative stress	Wang et al. (2024)
MLN4924	NAE	/	Regulating inflammation and fibrosis	Hao et al. (2019)
VLX1570	USP14	/	Regulating cell apoptosis	Wang J. et al. (2022), Bazzaro and Linder (2020)
PR-619	Broad spectrum DUBs inhibitors	/	Regulating inflammation, fibrosis and oxidative stress	Altun et al. (2011)

### 8.1 LDN-57444 — UCHL1

Ubiquitin C-terminal Hydrolase L1 (UCHL1) is a deubiquitinating enzyme that participates in various disease processes by regulating the stability of substrate proteins. In the cardiovascular system, UCHL1 is believed to regulate inflammation and fibrosis through ubiquitination (Lei et al., 2020; Gong et al., 2021; Zhang Z. et al., 2021). A study by Tang HY et al. investigating the role of UCHL1 in pulmonary arterial hypertension (PAH) demonstrated that UCHL1 promotes pulmonary vascular remodeling by inhibiting the ubiquitination and degradation of AKT1, suggesting that UCHL1 might participate in atrial remodeling through similar mechanisms (Tang et al., 2024). This cross-disease common mechanism provides theoretical support for targeting UCHL1 to treat AF. LDN-57444 is a selective UCHL1 inhibitor that modulates downstream signaling pathways by blocking its deubiquitinase activity. Currently, LDN-57444 has not been directly applied in clinical studies for AF, but its successful application in neurodegenerative diseases and cancer suggests its potential for cross-disease therapy (Liu et al., 2024), providing new ideas for targeted therapy of AF. Notably, Bi HL et al. found that LDN-57444 inhibition of UCHL1 attenuates Ang II-induced atrial fibrillation in mice (Bi et al., 2020). This provides a theoretical basis for clinical translation.

### 8.2 P22077 — USP7

P22077 is a cell-permeable small-molecule inhibitor targeting the ubiquitin-specific protease USP7 and its homologue USP47. Its core function is to inhibit deubiquitinase activity, thereby regulating tumor-related pathways. It has selective advantages, showing weak inhibitory activity against 14 other proteases (such as DEN1, SENP2), indicating high target specificity. Although research on P22077 is currently focused on the oncology field (Fan et al., 2013), its potential application in AF deserves exploration. Currently, studies by Gu YH and Wang Y et al. have demonstrated that at the animal level, P22077 administration can alleviate Ang II-induced atrial fibrillation in mice, providing a theoretical basis for the clinical translation of AF treatment (Wang et al., 2024; Gu et al., 2022).

### 8.3 Other small molecule drugs

In addition to the aforementioned drugs with validated efficacy in animal experiments for AF treatment, other drugs targeting the ubiquitination network with high relevance to AF treatment show research promise in the AF field:

MLN4924: MLN4924 is an inhibitor of NEDD8-activating enzyme (NAE) that blocks the activity of Cullin-RING ligase (CRL) E3s by inhibiting their neddylation modification. In the field of cardiovascular disease, research has revealed the potent cardioprotective effects of MLN4924. By enhancing cell viability, this compound can alleviate left ventricular systolic dysfunction, thereby limiting infarct size in myocardial ischemia/reperfusion (MI/R) mice (Zhang J. et al., 2021), which can induce cardioprotective effects and inhibit factors contributing to AF.

VLX1570: Targets USP14 (a deubiquitinating enzyme). Similar to P22077, research is mainly concentrated in the field of cancer therapy. It inhibits DUB activity, leading to abnormal protein clearance and proteasome stress. Its potential application in AF is also worth exploring (Bazzaro and Linder, 2020).

PR-619: A broad-spectrum DUBs inhibitor. Mikael Altun et al. screened a library of USP7 activity modulators based on small-molecule diversity using a validated, robust *in vitro* assay (Ub-CHOP reporter system) for high-throughput screening. PR-619, one of the two hits confirmed in this screen, exhibited broader inhibitory effects compared to P22077, targeting multiple DUBs. Given the potential shown by P22077 in vivo experiments, PR-619 is also a promising research drug, while tissue specificity issues need to be addressed (Altun et al., 2011).

### 8.4 Targeted protein degradation for atrial fibrillation therapy

A single ubiquitinating or deubiquitinating enzyme is capable of interacting with multiple protein substrates, thereby governing a wide spectrum of biological processes. As noted earlier, USP38 has been shown to modulate multiple biological processes implicated in the pathogenesis of atrial fibrillation. Accordingly, achieving precise control over the substrate binding of ubiquitinating and deubiquitinating enzymes represents a critical challenge in the development of small-molecule therapeutics targeting these enzymes.

Recently, targeted protein degradation (TPD) has emerged as a highly promising therapeutic strategy. This approach employs engineered molecules that recruit target proteins to the cellular degradation machinery, thereby facilitating selective protein degradation (Baker et al., 2025). Proteolysis-targeting chimeras (PROTACs) represent a prototypical example of this technology. PROTACs are molecules that allow direct knockdown of proteins via the proteasomal degradation pathway. Traditional targeted drugs require strong binding affinity to the target protein, whereas drugs like PROTACs can mark the target protein for degradation even with weaker binding, potentially providing solutions for 80% of undruggable targets (Fang Y. et al., 2023; Han and Sun, 2020). PROTACs are heterobifunctional molecules consisting of two ligands connected by a linker: one for recruiting and binding the target protein, and the other for recruiting and binding an E3 ubiquitin ligase (Joshi et al., 2023). Designing bifunctional molecules to specifically recruit pathogenic proteins to E3 ligases for degradation offers a future direction for achieving ultra-high specificity in AF treatment.

Nanotechnology represents another novel TPD strategy. Nanoparticle-mediated TPD (NanoTPD) utilizes nanomaterials as a multifunctional platform to trigger targeted protein degradation by bringing target proteins and E3 ubiquitin ligases into close proximity. Compared with PROTACs, NanoTPD overcomes the limitations of small molecules and exhibits superior performance in membrane protein targeting, tissue delivery, and application flexibility (Baker et al., 2025).

Darko-Boateng and colleagues developed a novel nanomaterial, divalent nanobodies (DiVas), for the precise modulation of ion channel ubiquitination. Neural precursor cell-expressed developmentally downregulated gene 4–2(NEDD4-2), a member of the HECT family of E3 ubiquitin ligases, is a key regulator of the function of multiple ion channels, many of which are implicated in the pathogenesis and progression of atrial fibrillation (Goel et al., 2015; Grunnet et al., 2012; Darko-Boateng et al., 2025). By engineering NEDD4-2-targeted DiVas, the research team achieved effective and selective inhibition of a panel of ion channels including Cav2.2 and KCNQ1, with no significant effects on non-targeted ion channels (Darko-Boateng et al., 2025). In contrast to the direct overexpression of NEDD4-2, these DiVa nanobodies enable more precise targeting of specific ion channels and exert minimal perturbation on the core physiological processes of cells. This study thus presents a novel targeted intervention strategy and technological paradigm for atrial fibrillation therapy: the precise modulation of ion channel activity via the specific downregulation of the functional expression of core ion channels associated with atrial fibrillation. It is expected to overcome the limitations of traditional small-molecule drugs, such as poor targeting ability and prominent off-target side effects, thereby offering a highly promising new direction for the development of precision therapy for atrial fibrillation.

## 9 Discussion

The pathogenesis of atrial fibrillation (AF) involves complex molecular networks. Ubiquitination serves as a central hub linking atrial remodeling and arrhythmias by regulating multiple pathways, including inflammation, calcium homeostasis, ferroptosis, oxidative stress, and autophagy. Among these regulators, USP38, a key deubiquitinating enzyme (DUB), acts as a cross-pathway modulator: it promotes inflammatory responses by deubiquitinating NF-κB and enhancing the NF-κB/NLRP3 positive feedback loop, and stabilizes IRP2 to induce iron overload and ferroptosis in diabetic AF. This multifaceted role of USP38 suggests that targeting a single ubiquitin-related enzyme may simultaneously intervene in multiple pathological processes, breaking the “AF begets AF” vicious cycle caused by overlapping remodeling mechanisms. Furthermore, the TRIM21–NF-κB/Nox2 axis mediates oxidative stress after myocardial infarction, while the CREB1/USP9X/METTL3/PCDHGA10 axis regulates calcium handling. Collectively, these findings confirm that ubiquitination modulates AF progression through pathway-specific E3 ligase-DUB substrate interactions. Among the differentially expressed ubiquitination-related genes identified by RNA-Seq, KBTBD13 emerges as a promising candidate. Highly expressed in cardiomyocytes, KBTBD13 mutations are associated with nemaline myopathy type 6 (NEM6). Notably, families carrying KBTBD13 mutations exhibit significantly higher rates of cardiomyopathy and AF compared to the general population. Combined with its known functions, KBTBD13 may contribute to AF pathogenesis by directly affecting cardiomyocyte function.

This review systematically summarizes the critical role of ubiquitination in AF and its potential therapeutic strategies. Unlike previous studies focusing on individual pathways, this work integrates six core pathological processes, revealing the synergistic and interactive nature of ubiquitination regulation—such as crosstalk between oxidative stress and inflammation, and links between autophagy and calcium dysregulation. This integration provides a theoretical basis for “multi-target synergistic intervention.” We highlight promising candidate drugs: LDN-57444 (a UCHL1 inhibitor) and P22077 (a USP7 inhibitor) have been validated in animal models to alleviate Ang II-induced AF by regulating inflammation, fibrosis, and oxidative stress; MLN4924 (a NAE inhibitor) exerts cardioprotective effects by suppressing atrial remodeling; PROTACs (proteolysis-targeting chimeras), an emerging technology that degrades pathogenic proteins by recruiting E3 ligases, offer a novel therapeutic solution. NanoTPD uses nanomaterials as a multifunctional platform to trigger targeted protein degradation by bringing target proteins and E3 ubiquitin ligases into close proximity. These findings bridge the gap between basic research and clinical application, laying the foundation for developing novel AF therapies beyond traditional rate/rhythm control and anticoagulation.

Despite significant progress, several bottlenecks hinder the translation of ubiquitination research into clinical practice. First, the specificity of ubiquitination regulation remains incompletely understood: how do enzymes like USP38 selectively recognize different substrates (e.g., NF-κB, IRP2, STRAP) under distinct pathological conditions (e.g., pressure overload, diabetes, chronic kidney disease)? Second, tissue specificity of drugs is a critical concern: broad-spectrum DUB inhibitors such as PR-619 may disrupt normal cellular functions in non-cardiac tissues, leading to off-target effects. Third, clinical heterogeneity must be addressed: the expression profiles and regulatory roles of ubiquitin-related enzymes may vary across AF subtypes (paroxysmal vs. persistent, valvular vs. non-valvular), requiring large-scale clinical cohorts to validate potential biomarkers. Additionally, autophagy exerts a dual role in AF—protective under physiological conditions but pathogenic when overactivated—posing a challenge for therapeutic modulation: interventions must precisely control autophagic flux rather than simply inhibiting or activating it.

The ubiquitination regulatory network offers a new dimension for AF treatment, from molecular mechanisms to clinical translation. Future research should focus on deciphering the spatiotemporal dynamics of ubiquitination, developing tissue-specific drugs, and designing multimodal combination strategies to transition from symptom control to mechanism-based intervention. Only through interdisciplinary collaboration—integrating fields such as nanomaterials, artificial intelligence, and clinical electrophysiology—can we overcome existing bottlenecks and provide more effective and safer therapeutic options for AF patients.

## Glossary

AB: aortic banding
AERP: atrial effective refractory period
AF: Atrial fibrillation
AMAMs: adult mouse atrial myocytes
Ang II: angiotensin II
CKD: chronic kidney disease
Co-IP: Co-immunoprecipitation
CRL: Cullin-RING ligase
Cx40: Connexin 40
Cx43: Connexin 43
DEGs: differentially expressed genes
DFO: deferoxamine
DiVas: divalent nanobodies
DUBs: deubiquitinating enzymes
IRP2: Iron Regulatory Protein 2
IS: indoxyl sulfate
LA: left atrium
MF: myocardial fibrosis
MI: myocardial infarction
MI/R: myocardial ischemia/reperfusion
NAE: NEDD8-activating enzyme
NanoTPD: Nanoparticle-mediated TPD
NEDD4-2: Neural precursor cell-expressed developmentally downregulated gene 4-2
NEM6: nemaline myopathy type 6
NLRP3: NOD-like receptor protein 3
NMAMs: neonatal mouse atrial myocytes
PAH: pulmonary arterial hypertension
PROTACs: Proteolysis-Targeting Chimeras
PTMs: post-translational modifications
RFP2: Ret Finger Protein 2
RNA-Seq: RNA sequencing
ROS: reactive oxygen species
RYR2: Ryanodine Receptor 2
SERCA2a: SR Ca-ATPase 2a
TAK1: transforming growth factor-β-activated kinase 1
TPD: targeted protein degradation
TRAF6: TNF receptor associated factor 6
TRIM21: Tripartite motif-containing protein 21
Ub: Ubiquitin
UBDs: Ub-binding domains
UBPs: Ub-binding proteins
UCHL1: Ubiquitin C-terminal Hydrolase L1
UPS: ubiquitin-proteasome system
USP38: Ubiquitin-specific protease 38
USP38cko: USP38 knockout
USP38-TG: USP38 transgenic
USP9X: Ubiquitin-specific peptidase 9 X-linked
WWP1: WW domain containing E3 ubiquitin protein ligase 1
WWP2: WW domain containing E3 ubiquitin protein ligase 2.
